# The Use of Complementary and Alternative Medicine among Patients with Inflammatory Bowel Disease Is Associated with Reduced Health-Related Quality of Life

**DOI:** 10.1155/2016/6453657

**Published:** 2016-11-27

**Authors:** Randi Opheim, Marte Lie Høivik, Tomm Bernklev, Lars-Petter Jelsness-Jørgensen, Bjørn Moum

**Affiliations:** ^1^Department of Gastroenterology, Oslo University Hospital, Postboks 4959 Nydalen, 0424 Oslo, Norway; ^2^University of Oslo, Institute of Health and Society, Postboks 1072 Blindern, 0316 Oslo, Norway; ^3^Telemark Hospital Trust, O&U, 3710 Skien, Norway; ^4^Institute of Clinical Medicine, University of Oslo, Postboks 1072 Blindern, 0316 Oslo, Norway; ^5^Health Science, Østfold University College, Postboks 700, 1757 Halden, Norway; ^6^Department of Gastroenterology, Østfold Hospital Trust, Sarpsborg, Norway

## Abstract

*Background and Aims.* Complementary and alternative medicine (CAM) use among patients with inflammatory bowel disease (IBD) is common. The aim of this study was to explore associations between CAM use and health-related quality of life (HRQoL) as well as identifying whether sociodemographic factors, disease activity, and personal resources (self-efficacy) influence HRQoL scores in users and nonusers of CAM.* Methods.* Measures included sociodemographic and disease-related data, the International-CAM Questionnaire, and General Self-Efficacy Scale. A univariate analysis of variance was used to assess the association between CAM use and HRQoL. The associations between clinical, demographic, and personal factors and HRQoL were examined through linear regression analyses.* Results.* CAM users had statistically significant lower SF-36 scores compared to nonusers and the background population. Nonusers scored significantly lower compared to the background population in two out of the eight SF-36 dimensions. Independent of CAM use, disease activity had a negative impact and self-efficacy had a positive impact on HRQoL.* Conclusions.* HRQoL in CAM users with IBD was significantly lower compared to nonusers and the background population. Independent of CAM use, disease activity was negatively associated with HRQoL. Self-efficacy had a positive impact on the mental health dimensions in both CAM users and nonusers.

## 1. Introduction

Inflammatory bowel disease (IBD), ulcerative colitis (UC), and Crohn's disease (CD) are chronic relapsing remitting diseases of the gastrointestinal tract. The course of IBD is often unpredictable. Some patients experience indolent disease and rare relapses while others develop severe intestinal inflammation and need long term immunosuppressive medication and surgery. IBD patients experience various symptoms throughout their disease course [1]. Some symptoms are directly related to disease activity, while others may be a consequence of the medical therapy. The disease imposes a considerable burden and significantly impacts patients functioning and health-related quality of life (HRQoL) [2].

Previous studies have reported that one out of two IBD patients turns to complementary and alternative medicine (CAM) [3–6]. CAM includes a broad range of therapies and products that are not generally considered to be a part of conventional medicine or integrated into the main healthcare system, such as acupuncture, homeopathy, herbal medicine, relaxation techniques, and meditation [7]. The diversity of CAM modalities may explain why the reported prevalence of CAM use ranges from 31% to 74% in IBD populations across Europe, North America, and Australia [5, 8–11].

Studies have shown that CAM use in IBD is influenced by health beliefs [8, 12, 13]. IBD patients have reported using CAM to improve HRQoL and wellbeing [8]. Important mediators for CAM use include symptom relief and the amelioration of adverse reactions to conventional medicine as well as the presence of comorbidities [14]. These results highlight that personal as well as disease-related factors influence the IBD patients' motivation to use CAM. Studies assessing HRQoL and CAM use in IBD populations have reported conflicting results. One study found an independent association between CAM use and reduced emotional functioning [12]. In contrast, two studies reported that CAM users had higher HRQoL scores compared to nonusers [13, 15]. The majority of studies, however, have found comparable HRQoL scores between CAM users and nonusers [5, 6, 16].

The primary aim of this study was to investigate potential associations between CAM use and HRQoL in patients with IBD. The secondary aims were to identify whether sociodemographic factors, disease activity, and personal factors influence HRQoL scores in users and nonusers of CAM.

## 2. Materials and Methods

### 2.1. Study Design

In this cross-sectional study, data related to the use of CAM, HRQoL, and self-efficacy, as well as clinical and sociodemographic factors were collected.

### 2.2. Study Population

Patients ≥ 18 years old with a previously verified IBD diagnosis (based on clinical, endoscopic, and histological findings) attending outpatient clinics at 14 hospitals in Norway were invited to participate. The inclusion period was from January 2009 to December 2011. The patients were asked to complete the self-administered questionnaire during a routine visit at the hospital.

### 2.3. Measurements

#### 2.3.1. Sociodemographic and Clinical Data

Sociodemographic variables included age, gender, educational level (12-year (secondary) education or less versus more than 12-year (college/university) education), marital status (married or cohabitating versus single, divorced, or partner living separately), work status (working, including being a student versus not working, including being a pensioner or work disabled), and smoking status (yes (defined as once or more daily) versus no).

Data concerning medical history and past surgical history for IBD were obtained from the patients' medical records. The Montreal classification system was used to classify disease location and behavior in CD and the disease extent in UC [17]. Disease activity was measured with the Harvey-Bradshaw activity index (HBI) in CD patients and the simple clinical colitis activity index (SCCAI) in UC patients [18, 19]. HBI scores >4 and SCCAI scores ≥3 were classified as active disease [20, 21]. Information regarding comorbidity and adverse drug reactions was self-reported by patients.

### 2.4. Questionnaires

#### 2.4.1. International CAM Questionnaire (I-CAM-Q)

The International CAM Questionnaire (I-CAM-Q) has been translated into Norwegian by the National Research Center in Complementary and Alternative Medicine (NAFKAM) [7]. The questionnaire contains the following sections: (1) CAM services, such as visits to alternative healthcare providers such as an acupuncturist, homeopath, spiritual/religious healer, chiropractor, reflexologist, kinesiologist, or laser treatment; (2) complementary treatments received from physicians; (3) CAM products, such as herbal medicine, homeopathic remedies, or dietary supplements; and (4) CAM self-help practices, such as relaxation techniques, visualization, yoga, meditation, Qigong, Tai Chi, prayer, or healing ceremonies. A CAM user was defined as someone who had (1) visited one or more alternative healthcare providers, (2) received complementary treatment(s) from a physician, (3) used herbal medicine or dietary supplements, or (4) used self-help practices within the previous 12 months.

#### 2.4.2. Short-Form 36 (SF-36)

To assess HRQoL, the generic Short-Form 36 (SF-36) was used. The SF-36 consists of 36 items and one multi-item scale for each of its eight conceptual domains. Higher scores indicate a better HRQoL. The domains are as follows: physical functioning (PF, 10 items), role limitations due to physical health (RP, 4 items), bodily pain (BP, 2 items), general health (GH, 5 items), vitality (VT, 4 items), social functioning (SF, 2 items), role limitations due to emotional problems (RE, 3 items), and mental health (MH, 5 items). The questionnaire has been shown to have a high validity and reliability [22]; it has been translated into Norwegian and validated in the general population in Norway [23]. Furthermore, it has previously been demonstrated that the SF-36 has satisfactory psychometric properties in the IBD population [24], and it has been used several times in Norwegian IBD populations [25–28]. The Norwegian SF-36 population reference was described by Loge and Kaasa [23] for a sample that was selected at random and retrieved from the National Population Register. The response rate was 67% (2323 persons). From this reference group, we use overall scores, which were adjusted for age, gender, and level of education.

#### 2.4.3. General Self-Efficacy Scale (GSE)

Personal resources were measured with The General Self-Efficacy Scale (GSE) [29]. The questionnaire measures a person's optimistic self-beliefs about coping with the demands of life. The GSE consists of 10 statements to which the respondents rate from 1 “completely agree” to 4 “completely disagree.” The GSE total score is calculated by summing each individual score (range 10 to 40). A higher score indicates stronger self-efficacy. The GSE has high reliability and validity [30] and has been translated into several languages, including Norwegian [31]. The internal consistency of the GSE scale in the present sample is *α* = 0.92.

### 2.5. Statistical Analysis

Univariate associations were assessed using Student's *t*-test for continuous data and Pearson's chi-square test for categorical data. When comparing SF-36 mean dimensional scores between CAM users and nonusers, we performed an analysis of covariance to adjust scores for age, gender, and level of education. The results are shown as estimated marginal means with 95% confidence intervals. The comparison of the dimensional scores between the background population and patients with IBD was performed with *Z*-scores (*Z*-score = the mean patient score minus the mean population score divided by the population standard deviation (SD)). Scores higher than zero indicate higher dimensional scores, and those lower than zero indicate lower dimensional scores in the patient population compared with the background population. *Z*-scores were evaluated with Cohen's effect size index (0.2 indicates no difference, 0.2−0.5 indicates a small difference, 0.5–0.8 indicates a moderate difference, and 0.8 indicates a large difference) [32]. Multiple linear regression analysis was used to assess the impact of sociodemographic factors, disease activity, and self-efficacy on HRQoL. In addition to gender and age, variables with a *p* value of <0.20 in bivariate analyses were entered into a multiple regression model. Cronbach's alpha was used to assess internal consistency of the GSE scale [33]. The level of significance was set at 0.05. All statistical analyses were performed with IBM SPSS Statistics for Windows, version 22.0 (IBM Corp. released 2013, Armonk, NY: IBM Corp.).

## 3. Ethical Considerations

The Regional Committees for Medical and Health Research Ethics in Norway (reference number: s-00858b) and the internal data protection officer at Oslo University Hospital approved this study. All patients received verbal and written information about the study prior to providing written informed consent.

## 4. Results

Of the 460 initial participants that provided written informed consent, 30 did not return the questionnaires after one reminder. Respondents with more than 50% missing items on the SF-36 scale and general self-efficacy scale (GSE) were also excluded from the analyses. For those with less than 50% missing items, the values were replaced by means of the items with valid responses. After controlling the questionnaires for missing values, 195 questionnaires were excluded due to incomplete responses, leaving the total number included in analyses at 235 (54.6%). There were no significant differences between those included in the analyses and those with nonevaluable questionnaires with regard to diagnosis, gender, age, or disease duration (Table 1).

Sociodemographic and clinical characteristics of the study population are shown in Table 2. In the overall IBD population, 116 (49.4%) were women, the median age was 39 years (range 18–79), and 112 (47.7%) had a higher education level. The majority of patients (*n* = 142, 60%) reported having active disease at the time of the study. One hundred and four (44.3%) had used some form of CAM in the last 12 months.

Sociodemographic and disease-related variables in CAM users versus nonusers are shown in Table 3. There was a higher proportion of women in the CAM user group compared to the nonuser group (60% versus 41%, *p* = 0.005). No statistically significant relationship between disease location and behavior in CD and disease extent in UC and CAM use was found (data not shown). The disease activity scores and medication regimens as well as self-efficacy scores were comparable in CAM users and nonusers.

### 4.1. HRQoL Scores

The SF-36 dimensional scores in CAM users and nonusers are shown in Table 4. Compared to nonusers, CAM users had statistically significant lower SF-36 scores after adjusting age, gender, and educational level in all dimensions except for role limitations due to emotional problems and mental health.

**Figure 1:**
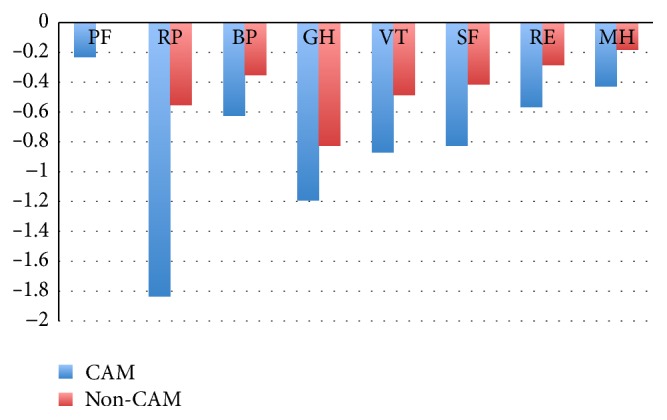
SF-36 scores in CAM users versus nonusers compared to the Norwegian background population. PF: physical function, RP: role physical, BP: bodily pain, GH: general health, VT: vitality, SF: social function, RE: role emotional, and MH: mental health. *Z* scores = mean patient score minus the mean population score divided by the SD of the population scores. Cohen's effect size index: 0.2, no difference; 0.2 to 0.5, small differences; 0.5 to 0.8, moderate differences; 0.8, large differences.

Compared to the Norwegian background population (Figure 1), CAM users had mean *Z*-scores between −0.5 and −0.8 in the dimensions of bodily pain and role limitations due to emotional problems representing a moderate reduction according to Cohen's effect size index. In the dimensions role limitation due to physical health, general health, vitality, and social functioning, the *Z*-score was beyond −0.8, indicating a large reduction in HRQoL compared with the reference population. Among nonusers, compared to the background population, the dimensions role limitation due to physical health and general health was moderately to largely different (*Z*-score = −0.5 to −0.8).

To assess the impact of relevant factors on HRQoL, we conducted linear regression analyses. The included variables were gender, age, education level, work status, relationship status, disease duration, disease activity, and self-efficacy (Table 5). The analyses were stratified by CAM use. The results showed that, independent of CAM use, disease activity was associated with a significant reduction in the SF-36 dimensional scores. Additionally, in CAM users, an older age was associated with a significant increase in the dimensions of vitality and mental health; furthermore, work status was associated with a significant increase in the dimensions of physical function and role limitations due to physical health and bodily pain. Self-efficacy was positively associated with the dimensions of role limitations due to emotional problems and mental health. In nonusers, work status was associated with an increase in the dimensions of physical function, role limitation due to physical health, bodily pain, general health, and social functioning. Self-efficacy was positively associated with the social function, role limitations due to emotional problems, and mental health dimensions.

## 5. Discussion

In this cross-sectional study of IBD outpatients, CAM users' HRQoL scores were significantly lower compared to those of nonusers and the general background population. Non-CAM users HRQoL scores were comparable with the background population for all dimensions except for role emotional and mental health. Independent of CAM use, disease activity was associated with reduced HRQoL scores.

The impaired HRQoL among CAM users observed in our study aligns with results from other disease groups such as arthritis, asthma, and cancer [34–36]. However, in studies of IBD populations, the results have been conflicting; the majority of studies have found comparable HRQoL scores between CAM users and nonusers [5, 6, 16]. The studies assessing the relationship between CAM use and HRQoL in IBD have deployed disease specific HRQoL questionnaires. In our study, we used a generic HRQoL questionnaire and were able to compare our results with the general background population. Compared to the background population, a significant reduction in 5 out of 8 dimensions was observed in CAM users. All five dimensions were of moderate to high differences according to Cohen's effect size index, thus defined as clinically relevant [32]. In contrast, the nonusers' HRQoL scores in our study are comparable with HRQoL scores in other IBD populations [37]. In particular, the only dimension with a *Z*-score beyond −0.8, indicating a large difference, was the general health dimension. This is comparable with results from the studies of other IBD populations, which have shown that the general health dimension has the largest reduction in HRQoL [37, 38]. The dimension of role limitation due to physical health in nonusers showed a significant reduction of a moderate difference (*Z* score −0.5) compared to a large difference (*Z* score –1.8) in CAM users. The questions asked in the role limitation due to physical health dimension include the following: (i) “Have you cut down the amount of time spent on work or other activities?”; (ii) “Have you accomplished less than would like?”; (iii) “Have you been limited in the type of work or other activities?”; and (iv) “Have you had difficulty performing work or other activities?” Consequently, it may be hypothesized that CAM users are those who feel limited by their disease in daily life.

Disease activity negatively influenced HRQoL independent of CAM use. It is well known that HRQoL is reduced in patients with active disease. A recent systematic review found that 10 out of 29 included studies reported a significantly negative relationship between disease activity and HRQL scores [39]. Other disease-related factors, however, such as extra intestinal complications, experience of adverse drug reactions, and comorbid conditions, have also been associated with CAM use [3, 8, 11, 13]. These results may indicate that patients seek CAM for problems that, from their point of view, cannot be resolved by conventional medicine alone. In a newly published study of Swedish IBD patients, the patients report an effect of CAM use on general health and wellbeing but no improvement in disease symptoms [4].

Increased self-efficacy (the strength of an individual's belief in their ability to cope with difficult demands in life) had a positive impact on HRQoL and, in particular, mental health in both CAM users and nonusers. Self-efficacy is a personal resource important for exercising personal control over motivation, behavior, and social environments, which seem to contribute positively to a person's HRQoL. Low personal control over illness and a belief that IBD may have serious consequences in previous studies predicted psychological stress, poorer HRQoL, and functional independence in IBD patients [40, 41]. One of the largest surveys on CAM use in IBD found that common reasons for CAM use was a need to obtain a greater control over one's own disease and take an active role in managing one's health and disease [14]. We were not able to control for other factors, such as how the IBD patients perceive their illness and its consequences (i.e., illness perception), which could have provided additional information about how these patients relate to their disease and their health [41]. In addition, in our study, we combined all forms of CAM into a single CAM use variable; thus, we cannot exclude that self-reported HRQoL varies among different types of CAM.

Several factors may explain the main finding in this study that CAM users reveal significantly lower scores in HRQoL compared to nonusers and the background population. The HRQoL dimensions most affected are the dimensions relating to vitality and physical and social functioning and roles. These dimensions concern how you experience yourself and your situation and how the disease affects you in general (general health). Our study shows that CAM users are affected in a stronger sense than nonusers in this manner. In this way, users of CAM might do so in an attempt to increase autonomy and a sense of control in their disease management.

In conclusion, HRQoL scores were significantly lower in CAM users compared to nonusers and significantly lower than in the background population. Nonusers had significantly lower scores compared to the background population on the general health and role limitation due to physical health dimensions. Independent of CAM use, disease activity was negatively associated and self-efficacy was positively associated with HRQoL.

## Figures and Tables

**Table 1:** Diagnosis, demographic, and clinical data for those included compared to those not included.

	Included (*n* = 235)	Not included (*n* = 192)	*p* value
*Diagnosis*			
Ulcerative colitis *n* (%)	112 (46)	88 (41)	0.387^*∗*^
Crohn's disease* n* (%)	134 (54)	124 (59)	
*Gender*			
Female *n* (%)	117 (49)	95 (50)	0.939^*∗*^
*Age*			
Median (range)	39 (18–79)	40 (18–73)	0.151^†^
*Disease duration*			
Median (range)	8 (0.10–45)	9 (1–44)	0.059^†^

^*∗*^ *χ* ^2^

^†^Mann Whitney *U* test.

**Table 2:** Comparison of sociodemographic and clinical characteristics of the study population.

	IBD (*N* = 235)	CD (*n* = 129)	UC (*n* = 106)	*p*value^*∗*^
*Gender *				
Female *n* (%)	116 (49.4)	72 (55.8)	44 (41.5)	0.029
*Age*				
Median (range) in years	39 (18–79)	37 (18–75)	41 (20–79)	0.034
*Education level *				
>12 years *n* (%)	112 (47.7)	59 (45.7)	53 (50.0)	0.515
*Work status *				
Working/student *n* (%)	156 (66.4)	80 (62.0)	76 (71.7)	0.118
*Marital status*				
Married or cohabitant *n* (%)	162 (68.9)	84 (65.1)	78 (73.6)	0.163
*CAM use*	104 (44.3)	49 (38.0)	55 (51.9)	0.033
*Current smoking*				
Yes *n* (%)	47 (20.0)	39 (30.2)	8 (7.5)	<0.001
*Disease duration*				
Median (range) in years	8 (0.1–45)	10 (0.1–38)	6 (0.2–45)	<0.001
*Disease localization*				
Proctitis *n* (%)			19 (19.2)	
Left sided *n* (%)			20 (20.2)	
Extensive *n* (%)			60 (60.6)	
L1 terminal ileum *n* (%)		22 (18.0)		
L2 colon *n* (%)		30 (24.2)		
L3 ileocolon *n* (%)		60 (49.2)		
L4 upper GI *n* (%)		10 (8.2)		
*Disease behavior*				
Inflammatory *n* (%)		36 (40.4)		
Stricturing *n* (%)		27 (30.3)		
Penetrating *n* (%)		26 (29.2)		
Perianal disease *n* (%)		22 (24.7)		
*Active disease* ^a^ *n* (%)	142 (60.4)	72 (69.0)	70 (56.0)	

IBD: inflammatory bowel disease; UC: ulcerative colitis; CD: Crohn's disease; CAM: complementary and alternative medicine.

__^a^Subjective reported active disease: simple clinical colitis activity index ≥3 in UC or Harvey-Bradshaw activity index >4 in CD.

Continuous variables were estimated by the Mann–Whitney *U* test, and chi-squared test (*χ* ^2^) was used to compare proportions.

^*∗*^ *p* value estimated between CD and UC.

**Table 3:** Differences in sociodemographic, disease related, and self-efficacy variables between CAM users and non-CAM users (*N* = 235).

	CAM users (*n* = 104)	Non-CAM users (*n* = 131)	*p*value
*Gender*			
Female *n* (%)	62 (60)	54 (41)	0.005
*Age*			
Median (range) in years	40 (20–79)	43 (20–78)	0.440
*Education level *			
>12 years *n* (%)	56 (54)	56 (43)	0.091
*Work status *			
Working/student *n* (%)	68 (65)	88 (67)	0.773
*Marital status*			
Married or cohabitant *n* (%)	70 (67)	92 (70)	0.631
*Current smoking*			
Yes *n* (%)	19 (18)	28 (21)	0.555
*Disease duration*			
Median (range) in years	8.0 (0.1–36)	8 (0.5–45)	0.732
*Active disease* ^a^ *n*%	78 (75)	26 (25)	0.104
*Self-efficacy *			
Mean (SD)	29.34 (5.48)	29.79 (6.16)	0.551

CAM: complementary and alternative medicine.

__^a^Active disease: simple clinical colitis activity index ≥3 in UC or Harvey-Bradshaw activity index >4 in CD.

Continuous variables were estimated by the Mann–Whitney *U* test, and chi-squared test (*χ* ^2^) was used to compare proportions.

**Table 4:** Mean SF-36 dimensional scores with 95% confidence intervals in IBD patients.

	All patients (*n* = 235)^a^	CAM users (*n* = 104)^a^	Non-CAM users (*n* = 131)^a^	*p*value^b^
PF	86 [84 to 89]	84 [80 to 87]	88 [86 to 91]	0.037
RP	55 [49 to 61]	47 [38 to 55]	61 [54 to 69]	0.011
BP	64 [61 to 67]	60 [55 to 65]	67 [63 to 72]	0.050
GH	55 [52 to 58]	51 [46 to 55]	59 [55 to 63]	0.008
VT	46 [43 to 49]	42 [40 to 47]	50 [45 to 53]	0.027
SF	73 [69 to 76]	68 [63 to 73]	77 [72 to 81]	0.009
RE	70 [65 to 75]	65 [57 to 73]	74 [67 to 81]	0.079
MH	75 [73 to 77]	72 [69 to 76]	76 [74 to 79]	0.065

IBD: inflammatory bowel disease; CAM: complementary and alternative medicine.

PF: physical function, RP: role physical, BP: bodily pain, GH: general health, VT: vitality, SF: social function, RE: role emotional, and MH: mental health.

^a^Adjusted for age, gender, and education.

^b^ *p* value estimated between CAM users and nonusers.

**Table 5:** Linear regression model for SF 36 dimensional scores in CAM users and nonusers.

	PF	RP	BP	GH	VT	SF	RE	MH
CAM users [*n* = 104]								
Age	−0.060 [−0.363 to 0.243]	0.287 [−0.417 to 0.991]	0.006^$^ [−0.408 to 0.420]	0.163 [−0.171 to 0.501]	0.325^*∗*^ [−0.004 to 0.653]	−0.046 [−0.486 to 0.394]	−0.157 [−0.831 to 0.517]	0.288^*∗*^ [0.016 to 0.559]
Gender [Women ref]	9.602^*∗*^ [2.017 to 17.187]	15.832 [−0.986 to 31.894]	9.144 [−0.605 to 18.892]	7.489 [−0.913 to 15.891]	9.460^*∗*^ [1.197 to 17.722]	8.449 [−2.626 to 19.524]	5.252 [−11.880 to 22.384]	1.469 [−5.105 to 8.043]
Education	—	15.132 [−1.630 to 31.894]	—	—	—	—	—	—
Work status	8.743^*∗*^	19.378^*∗*^	12.923^$^	6.497	—	—	—	—
[Not working ref]	[1.011 to 16.475]	[1.698 to 37.059]	[3.278 to 22.567]	[−2.068 to 15.061]				
Relationship status [Single ref]	—	—	3.792 [−6.322 to 13.905]	—	—	—	—	—
Disease-activity	−8.734^*∗*^ [−17.287 to −0.181]	−17.461^$^ [−36.011 to –1.088]	−19.128^$^ [−29.803 to −8.454]	−10.859^*∗*^ [−20.333 to −1.385]	−6.101 [−15.410 to 3.207]	−1.879 [−10.597 to 14.356]	7.700 [−11.273 to 26.672]	0.262 [−6.854 to 7.378]
Disease duration	—	1.054 [−0.066 to 2.173]	−0.427 [−1.066 to 0.212]	—	—	—	—	0.277 [−0.151 to 0.705]
Self-efficacy	—	—	−0.336 [−1.106 to 0.435]	—	—	—	1.455^*∗*^ [0.092 to 2.819]	0.980^$^ [0.465 to 1.495]

Non-CAM users [*n* = 131]								
Age	−0.005 [−0.226 to 0.215]	0.168 [−0.355 to 0.690]	0.300 [−0.031 to 0.630]	0.277 [−0.012 to 0.567]	0.209 [−0.091 to 0.509]	0.088 [−0.211 to 0.388]	0.030 [−0.478 to 0.539]	0.012 [−0.199 to 0.224]
Gender [Women ref]	−4.053 [−10.220 to 2.114]	−0.301 [−15.219 to 14.618]	0.522 [−8.923 to 9.967]	−4.264 [−12.320 to 3.792]	4.979 [−3.380 to 13.337]	3.372 [−4.965 to 11.709]	2.742 [−11.773 to 17. 258]	−0.237 [−6.123 to 5.649]
Education	1.448 [−4.744 to 7.640]	0.541 [−14.249 to 15.331]	2.868 [−6.496 to 12.232]	−0.404 [−8.347 to 7.539]	1.076 [−7.165 to 9.317]	1.760 [−6.460 to 9.981]	3.510 [−10.882 to 17.901]	2.471 [−3.333 to 8.275]
Work status [Not working ref]	9.664^$^ [2.951 to 16.377]	24.175^*∗*^ [8.230 to 40.120]	12.134^*∗*^ [2.093 to 22.229]	14.196^$^ [5.631 to 22.761]	7.919 [−0.986 to 16.805]	10.360^*∗*^ [1.469 to 19.224]	14.699 [−0.816 to 30.901]	2.512 [−3.746 to 8.770]
Relationship status [Single ref]	—	—	—	1.423 [−7.061 to 9.908]	5.114 [−3.689 to 13.917]	8.460 [−0.322 to 17.241]	—	4.069 [−2.131 to 10.268]
Disease activity	−11.872^$^ [−18.257 to −5.487]	−35.724^$^ [−50.924 to –20.525]	−25.649^$^ [−35.272 to −16.026]	−19.818^$^ [−27.991 to −11.646]	−20.062^$^ [−28.542 to −11.583]	−16.028^$^ [−24.486 to −7.570]	−18.027^*∗*^ [−32.816 to −3.238]	−9.643^$^ [−15.615 to −3.672]
Disease duration	—	—	—	—	—	—	—	—
Self-efficacy	—	0.699 [−0.593 to 1.992]	0.060 [0.514 to 1.123]	0.664 [−0.051 to 1.380]	0.578 [−0.165 to 1.320]	1.107^$^ [0.366 to 1.848]	1.464^*∗*^ [0.207 to 2.722]	0.942^$^ [0.419 to 1.464]

Linear regression model to estimate the effect of selected variables on SF-36 dimensional scores. The presented variables are estimated *B* with 95% confidence intervals.

^$^ *p* < 0.01; _ _^*∗*^ *p* < 0.05.

CAM: complementary and alternative medicine, PF: physical function, RP: role physical, BP: bodily pain, GH: general health, VT: vitality, SF: social function, RE: role emotional, and MH: mental health.
